# Automatic Recognition Method of Machine English Translation Errors Based on Multisignal Feature Fusion

**DOI:** 10.1155/2022/2987227

**Published:** 2022-05-12

**Authors:** Ruisi Zhang, Haibo Huang

**Affiliations:** ^1^School of International Studies, Hunan Institute of Technology, Hengyang, Hunan 421002, China; ^2^School of Electrical Information Engineering, Hunan Institute of Technology, Hengyang, Hunan 421002, China

## Abstract

The current automatic recognition method of machine English translation errors has poor semantic analysis ability, resulting in low accuracy of recognition results. Therefore, this paper designs an automatic recognition method for machine English translation errors based on multifeature fusion. Manually classify and summarize the real error sentence pairs, falsify a large amount of data by means of data enhancement, enhance the effect and robustness of the machine translation error detection model, and add the source text to translation length ratio information and the translation language model PPL into the model input. The score feature information can further improve the classification accuracy of the error detection model. Based on this error detection scheme, the detection results can be used for subsequent error correction and can also be used for error prompts to provide translation user experience; it can also be used for evaluation indicators of machine translation effects. The experimental results show that the word posterior probability features calculated by different methods have a significant impact on the classification error rate, and adding source word features based on the combination of word posterior probability and linguistic features can significantly reduce the classification error rate, to improve the translation error detection ability.

## 1. Introduction

Machine translation refers to a technology that, given a language, uses a certain model or algorithm to translate it into another language that maintains the same semantic information. As a key task in the field of natural language processing, machine translation has played an important role in the communication between countries and nations in recent years. Machine translation is the study of the automatic conversion of one natural language (source language) to another natural language (target language) by means of computers. Machine translation first originated from the idea of using cryptography to solve the task of human language translation proposed by American scientist Weaver in 1949. In 1954, IBM and Georgetown University used electronic computers to translate a few simple Russian sentences into English for the first time. The translation system contains 6 translation rules and 250 words. This experiment demonstrated the process of machine translation using a dictionary and translation rule-based method. Although it was only a preliminary success, it has since aroused the research fever of machine translation in research institutions in the Soviet Union, the United Kingdom, and Japan, and greatly promoted the research progress of early machine translation. However, in a report entitled LANGUAGE AND MACHINES (Language and Machines) published by the American Automatic Language Processing Advisory Council in 1966, machine translation was completely rejected, which led to the subsequent whole industry and academia began to avoid machine translation. Translation studies suffer from this. Historically, many artificial intelligence research, including machine translation, suffered certain setbacks in that era. The main reason was that the technical level at that time was still relatively low, and the people had high expectations for machine translation and other technologies. Until the mid-to-late 1970s, the exchanges between countries became increasingly close, the communication barriers formed between different languages became more and more serious, and people's demand for machine translation became more and more intense. At the same time, the development of corpus linguistics and computer science became machine translation that offers new possibilities. Since then, machine translation has entered a period of rapid development. After decades of evolution, it has formed three stages: rule-based machine translation, model-based statistical machine translation, and deep learning-based neural machine translation [1–8]. The transformer framework based on attention mechanism is very effective in translation tasks.

With the more and more frequent exchanges among the various ethnic groups in the world, there is an increasingly urgent need for machine translation to solve the problem of language barriers between various ethnic groups. Machine translation is a project with huge economic and social benefits. Machine translation research requires solving a series of fundamental linguistic problems. The ideal goal of machine translation research is to be practical in a wide range of fields with a high rate of accurate automatic translation. The main obstacle to realizing this ideal goal is the lack of precise natural linguistics systems, description methods, and description tools. To study various linguistic phenomena and laws in natural language, it is particularly necessary to describe language knowledge correctly. The study of natural language is not only important for automatic translation (written, voice, and computer) between natural languages but also for information processing related to the understanding of natural language knowledge levels: automatic summarization, text shaping, automatic formation of practical office letters, information filtering, information selection, network integration services, and other fields are also very important [9–15].

It has been recognized that the acquisition of high-quality machine translation results requires analysis and understanding of the semantics of natural language, and semantic analysis and syntactic analysis should not be disconnected. Therefore, establishing and perfecting the grammar theory suitable for natural language analysis and generation is still a subject of exploration [16]. Some of the existing MT theories involve lexical analysis, some involve syntactic analysis, and some involve semantic analysis. Each has different starting points, different emphases, and each has its own characteristics, and each project has its own limitations. The research method of this paper is mainly based on semantics, based on semantic language theory, combined with syntactic and grammatical analysis, and inherits and develops the existing MT theory.

At present, the research of language technology lags behind computer technology. We should make more efforts in the formal description of language, in terms of semantic description, and establish a rich and complete description of the concept represented by the word, revealing the concept of the concept. A commonsense knowledge base of relationships between concepts and attributes. In the study of English which is easily misunderstood and mistranslated, this paper explores the formal description of language from the perspective of unified semantics (rather than from the perspective of a natural language) [17–20]. The syntactic-semantic tree and syntactic-semantic type tree proposed in this paper are concept hierarchy, which can reveal the syntactic and semantic relationship between concepts. The computer's English translation ability directly affects the application effect of translation results and is closely related to people's economic activities. However, there are grammatical errors in the English translation results, which make the computer translation results deviate and affect the output and judgment of the English translation results. Therefore, in previous studies, a large number of experts and scholars have proposed automatic identification methods for machine English translation errors, in an effort to reduce the impact of English translation errors on economic activities.

It has become the mainstream machine translation technology in the industry. Machine translation is basically available in general scenarios. Syntactic structure analysis refers to the process of judging whether the composition of a given input sentence conforms to the given grammar and analyzing the syntactic structure that conforms to the grammar rules. Generally speaking, syntactic structure analysis methods can be divided into two categories: rule-based analysis methods and statistics-based analysis methods. The rule-based syntactic structure analysis method uses hand-written grammatical rules to build a grammatical knowledge base, and at the same time uses conditional constraints and checking methods so that ambiguity and supergrammatical phenomena in sentences can be effectively dealt with. However, the workload is huge, and the knowledge bases in different fields are quite different, which is not conducive to the transfer of rules between fields. In addition, the analysis effect of this method is not good for long and difficult sentences. Therefore, it is necessary to study a more accurate automatic recognition method for machine English translation errors [21–23].

**Figure 1:**
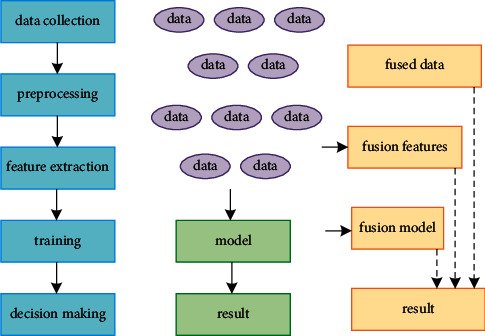
Multiple feature fusion levels.

Information fusion is to integrate information from different sources and remove redundancy, and apply the obtained fusion information to various subsequent tasks. As shown in Figure 1, information fusion can be divided into three levels: data fusion, feature fusion, and decision fusion. With the rapid development of deep learning technology, the wide application of distributed processing technology, and the substantial improvement of computer processing capabilities, the advantages of feature fusion are becoming more and more obvious. Feature fusion is widely used in the field of images, and it is also applicable in the field of text similarity calculation. Fusion of features extracted in different ways is an important means to improve the performance of the model. The underlying features contain more detailed information and are more noisy. High-level features have stronger semantic information and less ability to perceive details. How to efficiently integrate the two, take advantage of it, and get rid of its dross, is the key to improving the performance of the model [24, 25].

Many researchers improve the performance of the entire model by fusing multilayer features. According to the order of fusion and model prediction, they are divided into early fusion and late fusion. Early fusion is to first fuse the features of multiple layers and then use the fused features to train the model and only extract the features uniformly after complete fusion. This type of method is also called skip connection, that is, in a serial or parallel manner. The two classic feature fusion methods are as follows:

(1) Concat fusion, that is, serial feature fusion, which directly connects the two feature vectors. If the dimensions of the two input feature vectors *x* and *y* are *p* and *q*, then the dimension of the output feature vector *z* is *p* + *q*;
(2) The add mode fusion, that is, parallel feature fusion, combines two feature vectors into a complex vector. For the input features *x* and *y*, the fused feature is *z* = *x* + *iy*, where *i* is an imaginary unit. Late fusion is to improve model performance by combining the training results of different layers, start model training before completing the final fusion, and finally fuse multiple training results. There are two types of research ideas:
  1. The features are not fused, and the models are trained separately with multiscale features, and then the training results are synthesized
  2. The features are fused by pyramid network (PN), and the similarity is calculated after fusion

However, there are still many problems in data-driven neural machine translation technology, such as colloquial translation.

## 2. Multisignal Feature Fusion

In network flow classification, feature fusion can improve the accuracy. Low-rise and high-rise features each have their own strengths and weaknesses. Among them, the advantage of the former is that it contains rich information and has high discrimination ability; the disadvantage is that because it is very simple and does not undergo various transformations, it has lower semantics, and has a lot of noise at the same time; however, the latter is similar. On the contrary, in order to achieve the purpose of improving the classification performance, the most important point is to achieve the effective fusion of these two features.

Feature selection refers to picking out those parts from the existing features that are conducive to improving the prediction effect of the model, which is carried out before the training of the machine learning model. The problem of dimensional disaster may occur in stream classification tasks, and this link can effectively alleviate the dimensional disaster. Second, irrelevant features can be removed, reducing the difficulty of the classification task and making the model easier to understand. In addition, feature selection can remove irrelevant variables, minimizing the risk of overfitting. The feature selection process, subset search, evaluation methods, and common feature selection methods will be introduced below.

Feature fusion is very important to improve the accuracy of classification tasks. Feature fusion is divided into serial and parallel strategies. The former is to transform two or more features into one feature, which is suitable for fusing data of different dimensions. Since this fusion method does not need to unify the data dimension, it can effectively prevent the loss of information caused by the unification of the data dimension. The parallel strategy is to combine two feature vectors into a complex vector, suitable for models with the same output dimension. This method fuses different features without increasing the data dimension. Compared with the serial strategy, this method can effectively reduce the computational complexity. However, it needs to unify the data dimensions, which can lead to information loss. Errors, translation errors in professional fields, and low-resource language translation problems have not yet reached the ideal level under the current technical conditions, and the machine translation system will still output wrong translation results.

In image recognition, based on canonical correlation analysis, the transformation is achieved through the correlation between features, The resulting features will have higher correlations. However, the main limitation of CCA is that it ignores the relationships between classes. In order to overcome the shortcomings of CCA, a discriminant correlation analysis method is proposed, which not only retains the advantages of CCA but also increases the distinction between classes as much as possible. In order to reduce the loss of useful information to improve the classification accuracy, many methods have been proposed: fusion strategies through addition and L1 norm, fusion features from multiple feature extractors, through the combination of static and dynamic features, and through polynomial kernel functions feature fusion. A deep architecture-based feature generation model has been proposed, which improves the classification accuracy to a certain extent but requires a lot of data preprocessing time. While these feature fusion schemes provide better performance than single modality, they require separate feature extraction for each modality, resulting in longer processing time. Meanwhile, the fused features usually have high dimensionality, which increases the time overhead. In order to reduce the feature dimension, some people also convert the original features into semifeatures to maximize the mutual information between the transformed features and the target class. However, this approach may result in insufficient feature information generated. Feature fusion synthesizes relevant information extracted from network traffic data and can be used to improve classification accuracy in many cases. Although some achievements have been made in applying feature fusion to network flow classification, there are still some problems, such as how to generate more discriminative features to improve classification accuracy and the increase of time complexity due to the introduction of feature fusion.

To sum up, how to generate or fuse more discriminative features to improve the performance of network traffic classification with lower time overhead is still a hot research topic.

### 2.1. Feature Extraction of Machine English Translation Information

In natural language processing, feature vectors are derived from text data and can reflect various linguistic characteristics of text data. This mapping from text data to concrete vectors is called feature representation and is done through a feature extraction model. Picking the right features is part of what makes a machine learning task successful, and deep neural networks alleviate the reliance on feature engineering, especially for linguistic data that exists in the form of a series of symbols, which need to be converted into a numeric vector using specific methods.

Features in text can be represented in a scalar or countable form. A scalar feature usually takes the value 0 or 1, depending on whether a certain condition occurs, such as when the word “cat” occurs at least 1 time in the text, the feature takes 1, otherwise, it takes 0. The value of a countable feature depends on the frequency of a given event, such as the number of times “cat” appears in the text as the feature value.

For a sentence, a paragraph or a piece of text, the features that can be considered are the number and word order of characters and words in the text. The bag-of-words (BOWs) process is a common process for extracting features from sentences and texts. This method abstracts words into basic elements by considering the number of each word as a feature. Since the existing translation error detection solutions have problems such as low efficiency, high cost, and waste of manpower, and have gradually been unable to meet the rapidly growing translation needs.

In addition, the statistical results of external information can also be combined to focus on those words that appear frequently in a given text but rarely appear outside. When using the BOW method, TF-IDF weights are mostly combined. For a piece of text *d*, which is part of the corpus, we denote each word *w* in *d* as a normalized result:

(1) $$\begin{array}{c} \frac{N_{d}\left(w\right)}{\sum_{w{\;}^{\prime }\in d}N_{d}\left(w{\;}^{\prime }\right)}. \end{array}$$

The TF-IDF weights are

(2) $$\begin{array}{c} \frac{N_{d}\left(w\right)}{\sum_{w{\;}^{\prime }\in d}N_{d}\left(w{\;}^{\prime }\right)}\times \;\log\frac{\left|D\right|}{\left|\left\{d\in D:w\in d\right\}\right|}. \end{array}$$

When considering words in sentences and texts, a directly observable feature of a word is its position in the sentence, and words and characters surrounding it can also serve as features. The closer it is to the target word, the richer the amount of information the word has relative to distant words. For example, the N-gram model uses the first n-1 word sequences to predict the probability of word *n*, which includes local contextual features and text structural features, and is better than the bag-of-words model in practical applications.

In this research, the feature extraction algorithm of English translation will be integrated to make full use of parallel corpus. The extracted features are combined with the translation results to obtain the information features of the machine English translation. Through literature analysis, it can be found that machine translation can be divided into two parts, namely, the translation of the source language into the target language and the translation of the target language into the source language. The two translation processes are identical and share the word vector parameter. We set the source language statement to

(3) $$\begin{array}{c} A=\left\{a_{1},a_{2},\ldots ,a_{n}\right\}, \end{array}$$

where *a*_*i*_ represents the word of the source sentence; the target sentence is

(4) $$\begin{array}{c} B=\left\{b_{1},b_{2},\ldots ,b_{n}\right\}, \end{array}$$

where *b*_*j*_ represents the word embedding code of the target sentence; C represents the length of the source sentence; D represents the length of the target sentence. It is assumed that the encoder and decoder used in this translation are constructed as a neural network structure. The main function of the encoder is to encode the source sentence A into a fixed vector E, and at the same time decode E to obtain the target sentence D. The integrated translation process can be expressed as P (B|A:*α*), and the calculation process of the above conditional probability is obtained by using the multiplication rule, as shown in formula (5).

(5) $$\begin{array}{c} P\left(B|A:\alpha \right)=\prod_{i=1}^{T}p\left(b_{j}|a,b_{1},b_{2},\ldots ,b_{n-1}\alpha \right). \end{array}$$

### 2.2. Multifeature Fusion Prediction of Machine English Translation

According to the extracted machine-English translation features combined with the translation automatic evaluation method, the machine-English translation results are predicted. Using the Pearson coefficient as a guiding factor, the translation results are initially analyzed, and the specific calculation process is set as formula (6):

(6) $$\begin{array}{c} l=\frac{oxy-oxoy}{\sqrt{\text{d}x\text{d}y}}. \end{array}$$

Among them, *o* represents the mathematical expectation of the translation result; *d* represents the variance. In general, the value of this formula is −1 or 1. When the calculation result has a high correlation, the value is close to 1, otherwise, it is close to −1. According to the above formula, taking into account the characteristics of machine translation translation, a penalty function is introduced in the process of information prediction so as to ensure that the translation preference will not affect the translation result. Then, there is formula (7):

(7) $$\begin{array}{c} ℑ-N=U^{*}\exp\sum_{i=1}^{n}\varepsilon_{i}\mathrm{lg}\text{precision}. \end{array}$$

Among them, N is the number of penalty factors; *ε*_*i*_ is the flip vector; st − 1 is the influence vector of the hidden state at time t − 1 on the sentence; *xhr* is the word count vector of the decoder; *kt* is the encoder vector, and xhk is the encoder's word count vector. *nt* represents the step vector, and xhn represents the maximum encoding length vector, which is mainly used to limit the encoding steps; *st* represents the influence vector of the hidden state at time *t* on the sentence, *kt* represents the error vector, *nt* represents the error vector magnitude, and *st* is the machine English translation main cause of error.

We use this formula to predict the machine translation result and determine the correct rate of this result. At the same time, the information with a low accuracy rate is obtained as a training group for translation error recognition, and a corresponding support vector machine is constructed to make a secondary judgment on this part of the information. For the binary classification problem, in order to obtain the final reliable prediction result, the training set is set as

(8) $$\begin{array}{c} \left(z_{i},y_{i}\right),i=1,2,\ldots ,n,z_{i}\in R^{n},y_{i}\in \left\{\pm 1\right\}. \end{array}$$

The classification plane can be expressed as

(9) $$\begin{array}{c} \left(q*z\right)+k=0. \end{array}$$

Here, *k* represents the penalized plane slope; *q* and *z* represent the length and width of the penalized plane, respectively.

According to formula (9), the samples are correctly distinguished, and the classification interval is maximized. The optimal classification result needs to meet the following conditions:

(10) $$\begin{array}{c} y_{i}\left[\left(q*z\right)+k\right]\geq 1. \end{array}$$

Building a support vector machine based on formula (10), this problem can be optimized as

(11) $$\begin{array}{c} \left\{\begin{array}{c} \min1/2\beta *\beta +G\sum_{i}ℑ,\\ \text{s.t}{\text{.y}}_{i}\left[\varphi \left(q*z\right)+k\right]\geq 1-ℑ. \end{array}\right) \end{array}$$

Among them, *G* represents the cost coefficient in the classification process; *φ* (·) represents the nonlinear transformation function in the judgment process; *i* represents the slack variable function. According to this formula, the final judgment formula can be obtained as follows:

(12) $$\begin{array}{c} f\left(z\right)=\sum_{i=1}^{m}\eta_{i}y_{i}H\left(z_{i},z\right)+k. \end{array}$$

### 2.3. Design of Machine English Translation Error Recognition Algorithm

**Figure 2:**
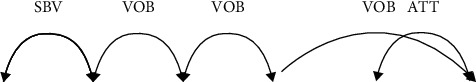
Directed graph of incorrect translation results.

In order to effectively fuse the semantic features and context structure interaction features extracted by the text semantic feature extraction module, text structure feature extraction module, and LSF feature extraction module, this paper studies different feature fusion methods, and compares different information fusion methods to help. According to the above setting results, a machine English translation error identification algorithm is designed to realize automatic identification of wrong translation. In order to make this algorithm feasible, the directed graph of wrong translation results is used as the main reference of the algorithm, and the directed graph of wrong translation is drawn, as shown in Figure 2.

In recent years, some researchers in this field have begun to try to utilize external knowledge sources as features, such as deep syntactic or deep semantic features. Word posterior probability features are based on system features and cannot provide sufficient knowledge of grammar or syntax. Therefore, it is considered to extract linguistic features such as syntax and semantics to improve the detection accuracy. This paper mainly uses two kinds of commonly used linguistic features: lexical features and syntactic features. A detailed description of these two types of features is given below.

For most of the words in the translation hypothesis, it is generally considered that the probability of a word sequence with high frequency and a sequence of part-of-speech tagging is higher than that of word sequence and part-of-speech tagging sequence with less frequency. Here, we considere the context information of the word and part-of-speech tagging, for each word and part-of-speech tagging, the first 2 and the last 2 and their own constitute feature vectors. Select a suitable feature fusion method to improve the accuracy of model calculation.

**Figure 3:**
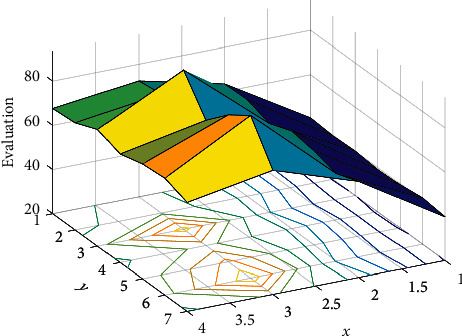
The evaluation.

Syntactic knowledge is generally obtained after syntactic analysis of the source language or target language by a syntactic analyzer. When the analyzer cannot analyze the entire sentence, the problem word is ignored to find the connection of the remaining words to complete the syntactic analysis. Words that are ignored become words that are not connected to other words in the sentence, called null-link words. It is generally believed that these words that are not related to other words are more likely to have grammatical errors, and this grammatical information is used to define binary syntactic features. The evaluation is shown in Figure 3.

## 3. Experimental Design

In order to confirm that the automatic recognition method of machine English translation errors based on multifeature fusion proposed in this study has application value, an experiment was constructed to analyze the application effect of this method. The statistical machine translation system in the experiment of this paper is a phrase-based machine translation system: Moses system. This system is utilized to output 10,000 best translation hypotheses for each source language sentence during the decoding process of the test set, i.e., *N* = 10000. The direction of the translation language pair is Chinese to English translation. The training corpus of the translation model is the dataset provided by LDC, with a total of 3,397,538 sentence pairs (mainly including Hong Kong news, FBIS, ISI Chinese-English network data, and Xinhua News). The language model adopts a five-element model, and its training corpus includes the English part of the above bilingual corpus and the Xinhua part of the English Gigaword, with a total of about 10 million sentences.

The development set of the machine translation system is the current set of the NIST machine translation evaluation task in 2006, with a total of 1,664 sentences, and each source language input corresponds to 4 reference answers. The test set is 1,082 sentences of NIST 2,005 current dataset and 1,357 sentences of NIST 2008 current data, and each sentence corresponds to 4 reference answers.

**Figure 4:**
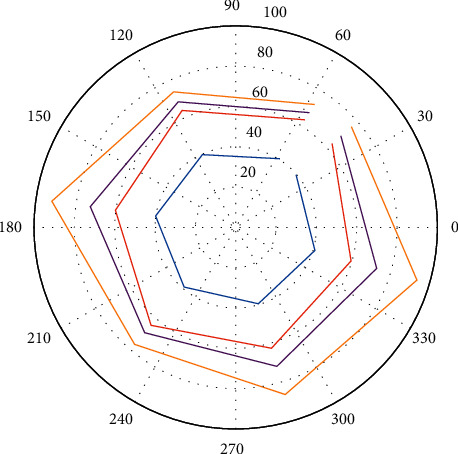
Radar figure.

Data annotation: This paper leverages the WER criterion from the TER toolkit to determine the true classification results for each word in the translation hypothesis. First, the reference translation is found with the smallest edit distance from the translation hypothesis among the four reference translations as the benchmark, and the WER criterion is used to align the hypothetical translation with the reference translation. If the word in the translation hypothesis is consistent with the word in the same position in the reference translation marked as *c*, otherwise marked as *i*. The radar figure is shown in Figure 4.

In the NIST MT 2008 translation results generated by the SMT system, the 1-best translation is assumed to contain 38,587 words, of which 14,658 are labeled *c* and 23,929 are labeled *i*, whose samples are the “correct” class ratio (RCW) is 37. 99%. The 1-best translation hypothesis of the NIST MT 2005 translation results contains 36,497 words, of which 15,179 are labeled *c* and 21,318 are labeled *i*, and the proportion of classes whose samples are “correct” (RCW) is 41. 59%. Dev and Test Sets: The NIST MT 2008 dataset (1 357 sentences) is used as the development set for maximum entropy model parameter training in the translation error detection task, and the NIST MT 2005 (1,082 sentences) dataset is used as the test set for the classification task.

**Figure 5:**
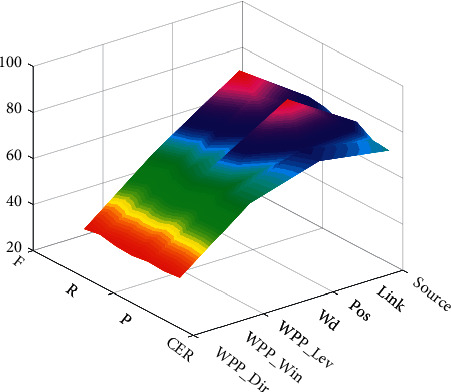
Results of individual feature for translation error detection.

The maximum entropy classifier is used to conduct independent feature classification experiments on three typical WPP features, three linguistic features, and extracted source word features, and analyze and compare the results. The results of the classification experiments are shown in Figure 5.

In Figure 5, WPP_Dir represents WPP based on fixed position; WPP_Lev represents WPP based on Levenshtein alignment, and WPP_Win represents WPP based on target position window. The word feature is represented by Wd, and the source word feature is represented by Source.

**Figure 6:**
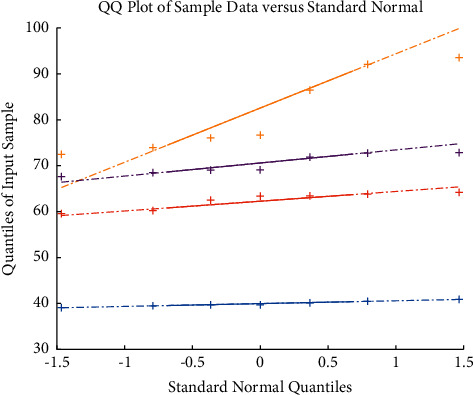
Prediction variation.

The CER of the baseline system is 41.59%. The following conclusions can be drawn from the data in Figure 6.

1. When the three typical WPP features are used independently, the CER value can be reduced to a certain extent, and the calculation method based on the target position window obtains a lower CER result than the other two methods because of its flexibility. WPP based on Levenshtein alignment performs best in terms of F-values.
2. Linguistic features perform better than standalone WPP features (except for Link). The word features can help the system find the error more accurately. The grammatical feature link has the highest recall rate, indicating that this feature can help the system find more errors. The data in Figure 6 also reveal the contribution of linguistic features to error detection, indicating that linguistic features can effectively reduce the classification error rate and improve the error prediction ability.
3. As far as CER is concerned, the source word features are reduced 4.59%. It proves the effectiveness of the source-side word features. It can be considered to extract richer source-side information as features and add them to the classifier to improve the performance of error detection and classification. The prediction variation is shown in Figure 6, which means the validation of the proposed when processing the machine English translation errors.

In the process of this experiment, the experimental platform is set as Windows and Linux system. In this system, the collection and processing of original translation information and extended information are completed, and the experimental part will be completed in Linux system. During the experiment, JAVA is used as the experimental control language, and the processing of files and the output of experimental results are controlled by this language. At the same time, we set the rules for merging experimental results, process the experimental results, and output the results.

The training data in the experiment mainly come from a laboratory database. The training dataset contains 5,000 wrong sentences and 5,000 corresponding correct sentences. These sentences are checked by all English-speaking staff who manually mark grammatical errors and correct each sentence. A mistake was obtained after combining this part of the information, and 10 experimental datasets were constructed, as shown in Figure 6.

**Figure 7:**
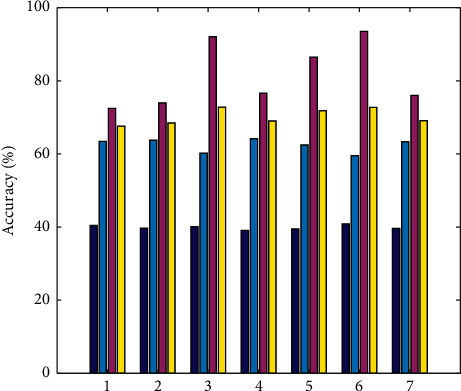
Accuracy.

**Figure 8:**
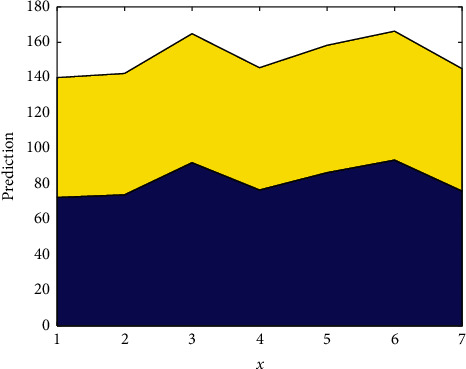
Prediction.

The collected translation information is divided according to the content in Figure 6, and the word vector is trained at the same time. We use the Word2vcc tool to train the translation information, set the vocabulary vector dimension of the translation information to 1024, set the window size to 10, use the negative sampling optimization algorithm to set the number of translation information samples to 10, and set the number of iterations to 20 times. In the experimental preparation stage, in order to ensure the reliability of the experimental results and reduce the error of the experimental results, the training set was trained using the translation information template preset in the previous research, and the training set was marked so as to divide and process the experimental data. The accuracy is also compared in Figure 7. Besides, the prediction is shown in Figure 8.

## 4. Conclusion

Aiming at the current English translation results, this paper proposes a new method for automatic recognition of translation errors, which has been proved to have certain practical effects through experiments. This time, the focus of the research is based on the accuracy of recognition, and there is no optimization for other fields. For this reason, other parts need to be analyzed in the follow-up research, and the shortcomings of this method should be improved and optimized to improve the translation effect and provide help for the development of machine translation technology.

Although the text similarity calculation model that combines various features proposed in this paper has achieved good performance, the model efficiency needs to be further optimized. In the future, the distributed computing framework can be combined with multiple graphics cards for parallel processing to improve the entire model of operating efficiency.

## Data Availability

The dataset can be accessed upon request.
